# Book Review

**Published:** 1933

**Authors:** 


### Reviews of Books

**The Medical Annual for 1933.** Edited by the late Carey F. Coombs, M.D., and A. Rendle Short, M.D., B.S., B.Sc. Pp. civ., 628. Illustrated. Bristol: John Wright & Sons, Ltd. 1933. Price 20s.—The interest of the present volume is indeed well maintained, for there is hardly a page which does not give a new suggestion as to treatment or describe some new form of disease. We entirely agree with the opinion quoted that "probably every doctor in active practice sees each year at least one case that can be better diagnosed or treated if he is aware of the advances on the subject recorded in recent numbers of the *Annual.*" Among the surgical novelties noticed are the removal of parathyroid tumours for osteitis fibrosa, the surgical treatment of detached retina and of pulmonary diseases by phrenicotomy. Many readers will be thankful for the complete table of doses for the New Pharmacopeia as given here, and for the summaries of recent work on the vitamins and on rheumatism. Moynihan's instructions to patients after gastric operations is another thing which we should like to keep by us always, especially with the amusing misprint which forbids patients to indulge in "high games" instead of high game as food. Attention is drawn to the startling results of the treatment of malaria by atebrin, and to the danger of aperients and even of enemata in pneumonia. The *Annual* has suffered a great loss by the untimely death of Dr. Carey Coombs, and we are sure that all readers will sympathize with the brief but affectionate tribute to his memory on page 8.

**Royal Berkshire Hospital Reports, 1932 and 1933.** Edited by H. S. Le Marquand, M.D. Illustrated. Reading. 1932 and 1933. Price 10s. 6d. each.—It is a new departure for a provincial hospital to publish annually a volume of collected papers, and the medical staff of the Royal Berkshire are to be congratulated, both on the form of these volumes and on the high standard of the contributions. The object of the *Reports* is to preserve permanent records of much useful information at present buried in the hospital notes, and secondly to supply the neighbouring practitioners with information relating to new methods of diagnosis and treatment. Many of the contributions will be of special interest to pathologists, whilst to the general practitioner we would especially commend Dr. John Mills' article on Glandular Fever (1932) and the very clear, simple and informative description of electrocardiograms and their interpretation by Dr. G. Lambert (1933). The next two volumes will be published together in 1935.

**The Thyroid Gland.** By C. R. HARINGTON, Ph.D., F.R.S. Pp. xiii., 222. Illustrated. London: Oxford University Press. 1933. Price 15s.—The avowed intention of the author of this volume is to give an account of the thyroid gland as it appears to the chemist: to do this it has been necessary to make incursions into the fields of physiology and pathology. Dr. Harington has given much time to researches on the thyroid gland, especially with regard to more satisfactory methods for the isolation of thyroxine, and, like all research workers, he has become thoroughly acquainted with the literature of his subject, and this acquaintance renders his monograph extremely valuable to all interested in the thyroid gland from various points of view. Although it is not written especially for clinicians, it should be of considerable use to physicians, since it contains an account of investigations on the thyroid gland from early times to the present day, and also an alphabetical list of authors who have dealt with this subject. It is interesting to observe that as far back as the thirteenth century goitre was treated by the administration of burnt sea sponge—some five or six hundred years before the isolation of the element iodine. The chapter on the chemistry of thyroxine is up to date, and should interest students of biochemistry, and the concluding chapter on the biological aspects of Graves's Disease should appeal to physiologists and practising physicians, and stimulate the latter to develop some satisfactory medical means of treating this condition. A number of interesting photographs are included to illustrate the effects of deficient thyroid secretion in human beings and in the lower animals. We congratulate both author and publisher on a treatise which should be of great value.

**Nervous Disorders in Infancy and Childhood.** By NEILL HOBHOUSE, M.D. Pp. viii., 212. London: H. K. Lewis & Co. Ltd. 1932. Price 8s. 6d.—The nervous complaints of children have not received as much attention in the medical literature of this country as has been afforded to them elsewhere, and Dr. Hobhouse has done a service to the practitioner in preparing this concise, lucid and readable volume. He describes most of the nervous syndromes which are met with in childhood in non-technical terms, so that even the practitioners who do not profess neurology may approach the book with confidence that they will not lose themselves in a sea of jargon. Naturally there are some points which will not meet with universal acceptance, such as his adherence to Stoeffel's operation for spastic paralysis (which produces such dramatic immediate improvement, but which is often attended by dire results in the form of obstinate contractures later on), but this is inevitable in any up-to-date medical work. The chapter on hyperkinesis is valuable inasmuch as he lays stress on the differentiation between tricks and tics and chorea proper, and makes it clear to the practitioner that while rest is essential for the latter it is the worst possible treatment for the former. There are two final chapters on psychoneuroses and mental defect which do not pretend to be adequate to their subject, but they are sufficient to indicate to the practitioner what he may be up against and may encourage him to seek further information elsewhere. Altogether a book to be commended.

**Paralysis in Children.** By R. G. GORDON, M.D., D.Sc., and M. FORRESTER BROWN, M.D., M.S. Pp. viii., 328. Illustrated. London: Oxford University Press. 1933. Price 15s.—This book has been written from the combined aspects of the neurologist and the orthopædic surgeon. This is a very sound idea, as each has so much to contribute towards the treatment of most cases of paralysis. It has been divided into three sections. The first outlines those general anatomical and physiological considerations without a due appreciation of which rational diagnosis and treatment are impossible. It contains many useful diagrams designed to refresh the memories of those of us whose recollections of the anatomy of the central nervous system have become a trifle hazy. The second section is considerably the longest. An account is given of each type of paralysis, which is then further illustrated by clinical records of cases. This section forms a most useful work of reference, and by itself fully justifies the publication of the whole book. The third part concerns treatment, and here the authors must have had a hard task to decide what to include and what to omit. On the whole they have exercised excellent discrimination by insisting upon principle rather than upon detail. For the latter some larger tome of orthopædic surgery must be consulted when necessary. The authors have deliberately repeated themselves in certain parts of the text, as is almost unavoidable in a work of this sort. A considerable number of the illustrations have also been duplicated, and this seems to the reviewer to be a mistake. If every illustration were exactly opposite the text it might be worth while, but this is not so. If one has to turn any pages the exact number makes little difference. As with all the Oxford Medical Publications this is a well-printed volume of handy size. It is well indexed.

**The Adjustment of Muscular Habits.** By JAMES K. McCONNEL, M.C. Pp. xi., 129. London : H. K. Lewis & Co. Ltd. 1933. Price 4s. 6d.—This little book is an extremely interesting one, and deals with a subject of great importance to all branches of the medical profession. During the last few years the attention of the profession, as well as of the public through the lay Press, has been drawn increasingly to the significance of postural defects—poor body mechanics, as our colleagues in the United States of America call them, but the chief stress has hitherto been laid on their mechanical aspects, somewhat to the neglect of the human or psychological side. It is evident that since the publication of his earlier book on *Shorter Convalescence* Colonel McConnel has been struggling with the problem, which confronts all workers in physical training, *i.e.* how to make the patient who feels comfortable in a poor attitude wish to replace it by a correct one, and how to convert a voluntary and temporary correction into a permanent, automatic balance. The author is obviously a successful practitioner in this art, and he endeavours in this book to impart the principles which he has found valuable in overcoming what is no easy problem. As a simple illustration of the psychological requirements, he points out that " one bite on an exposed dental nerve, or a cut in the pad of a finger may change abruptly the habits of a life-time," and also " our muscular behaviour varies with conditions, such as the stimuli, the environmental and internal circumstances." Accordingly " we must not confine ourselves to the motor aspect of habits, when we desire to substitute good muscular habits for bad ones." He further points out that many primary bad habits exist without giving rise to symptoms, due to the remarkable resistance of our bodily tissues, and only when resistance is reduced does disability occur, which causes most treatment to be directed to raising the resistance without correcting the faulty habit; whereas efficient treatment demands the elimination of both factors. A common example of this is the faulty postural and respiratory habit that follows recumbency after an abdominal operation, and which so seldom receives attention by either surgeon or family doctor. The author stresses the importance of enabling the patient to guide his own efforts by teaching him the mechanism of good and bad muscle actions and their effect on other systems of the body, and by assisting him to alter environmental conditions as far as possible, so that the initial achievement and its repetition may take place under conditions peculiarly favourable to it. Amongst external conditions *incentive* is one of the most important, and, of course, varies with different individuals. Another point often overlooked by gymnasts is that "the criterion of efficiency is *balanced action* of the numerous muscles, each in its allotted task." Another important consideration is that "rapidity in habit formation is found to depend on *avoiding direct* voluntary control and on inducing the required muscular responses in an indirect manner." The author draws attention to another aspect of the problem, frequently neglected, *i.e.* the interaction of various postural defects, so that it is rare for one to exist alone, and further that it is only by laying the responsibility for progress on the patient, instead of allowing him to rely on outside help, that we can avoid relapse, which is so common in these defects. Such are the main outlines of the author's thesis, and his book is well worth thoughtful perusal by members of every branch of the profession.

**Treatment of Rheumatoid Arthritis and Sciatica.** Second edition. By A H DOUTHWAITE, M.D. Pp. xii., 131. Illustrated. London: H. K. Lewis & Co. Ltd. 1933. Price 6s.—This book deals with the treatment of rheumatoid arthritis as distinct from septic arthritis and osteoarthritis. So much emphasis has been laid on the part played in the causation of the disease by septic foci that it is interesting to note that the author does not regard sepsis as the most important ætiological factor, and lays emphasis on the fact that at least equal attention must be paid to endocrine disturbances, vitamin deficiencies and metabolic changes. Amongst newer methods of treatment which are described attention should be paid to the use of arthrytin and allochrysine during the first stage, to the use of splints and of wax during the second stage, and the necessity for manipulative treatment during the third or non-progressive stage. In the portion of the book devoted to sciatica Dr. Douthwaite makes it evident that successful treatment must depend on correct diagnosis of the nature and site of the sciatic lesion, whether it be primary or secondary, peripheral or central. Most encouraging results are reported from epidural spinal injections and from spinal manipulation, and the author concludes that we should be able to look forward with confidence to a steady diminution of sufferers from chronic sciatica, which can now but rarely be described as intractable.

**Chronic Rheumatism and the Pre-rheumatic State.** By J. H. HINDLEY-SMITH, M.R.C.S. Pp. 154. London: H. K. Lewis & Co. Ltd. 1932. Price 5s. 6d.—This is a misleading and from its very simplicity a dangerous book. The author groups all chronic rheumatism together without any sort of clinical distinction. He states that the chronic rheumatic infection of childhood leads to an instable delicate condition in adolescence, and a state most closely resembling secondary rheumatoid arthritis in middle life, that these are all due to pharyngeal infection with a streptococcus, and that chronic rheumatism can be prevented and cured by a special regime, the chief features of which are vaccines and ultra-violet rays. The doses of vaccine suggested are considerably in excess of those recommended by recognized authorities at the present day. It is to be hoped that practitioners will not be misled by the convenient and simple panacea here presented, for chronic rheumatism is a complex and difficult subject, and the various clinical conditions included under this term are due to a variety of etiological factors, and consequently require a variety of therapeutic measures. There are no short cuts in medicine, and though the wish is often father to the thought, such wishes are not to be trusted.

**Diseases and Disorders of the Digestive Organs.** By ADOLPHE ABRAHAMS, O.B.E., M.D., F.R.C.P. Pp. vi., 110. London: John Bale, Sons and Danielsson Ltd. 1932. Price 2s. 6d.—This is a most excellent pocket monograph, illuminated with an occasional touch of humour, a welcome ray too infrequently shed on medical writings. Not that the subject is treated lightly; on the contrary, for its size the little book is very comprehensive. The diagnosis of gastric ulcer is rather weak: the merits of medical and surgical treatment are helpfully compared, and the all too frequent inadequacy of the former is very pointedly emphasized. The standard of time generally accepted as required for such should be readjusted ; the public and the profession as a whole are not alive to this as they are in respect of tuberculosis. Moreover, the farcical futility of attempting any ambulatory treatment for gastric ulcer is expressed, and with reason; "rather a patient on a comparatively generous diet but strictly confined to bed than prosecute the most elaborate procedures with diet and drugs whilst allowing physical activity." The probability of an ulcer diathesis, once an ulcer has been established, though healed, is very properly urged. The views expressed are wrapped in common sense : this is generally obvious and welcome in a well-balanced preamble to a discussion of the diagnosis of cancer of the stomach, and in a section devoted to nervous indigestion.

**Some Thoughts on Asthma.** By A. J. D. CAMERON, M.B., Ch.B. Pp. viii., 178. Illustrated. Bristol: John Wright & Sons Ltd. 1933. Price 7s. 6d.—Dr. Cameron's monograph on asthma gives his views on the production of the disease, and demonstrates the method of treatment carried out at the Sherwood Park Clinic at Tunbridge Wells. He describes it as the Haseltine treatment, and the book is full of his own experiences relating to the causation and treatment of the disease. The etiology of asthma is traced through its stages up to the final stage of bronchospasm. The fundamental origin is a basic toxicosis arising from the liver and intestines, these produce or are linked up with septic foci in the ethmoid region, or may be from teeth, gall-bladder, appendix, etc., which produce a state of irritability of the nervous system, a vagotonia which results in the attack of bronchospasm. Dr. Cameron describes the treatment of each stage. This is a monograph which will interest the medical practitioner. Though some of the treatment is best carried out at a recognized spa institution, in certain cases Dr. Cameron thinks it should be possible adequately to deal with it at home, provided the patient could be sent to a treatment centre daily.

**The Injured Workman.** By G. F. WALKER, M.D., M.R.C.P. Pp. xix., 190. Bristol: John Wright & Sons Ltd. 1933. Price 6s.—Most qualified men and women, whether in general or consultant practice, are called upon at one time or another to attend a court of law for the purpose of giving evidence in compensation cases. This may prove to be a difficult task for those who are not constantly doing this type of work, and may be a source of some anxiety. The book under review should be of the greatest value to the occasional medical witness, and solves many of his difficulties. There is a Foreword by Professor Maxwell Telling, which alone is most illuminating, the book itself being written in collaboration with four consultants, including a barrister-at-law. The opening chapter deals briefly with the law relating to the Workmen's Compensation Act, and advises the medical witness as to his conduct in court. This is followed by chapters on the more common accidents to workmen, their results and complications and warnings of the mistakes into which the unwary may be trapped. The subjects are dealt with systematically, injuries to the ear, nose, throat and eye are included. There are also a few words of advice on the all-important subject of malingering. The book is recommended with the utmost confidence.

**Practical Food Inspection.** Volume 2. By CHARLES R. A. MARTIN, M.R.S.I. Pp. vii., 249. London: H. K. Lewis & Co. Ltd. 1933. Price 10s. 6d.—This volume deals entirely with foods other than the meat of animals killed in slaughter houses, and with the first volume makes the work complete. It is intended not only for students qualifying to undertake the duties of meat and food inspectors, but also for meat and food inspectors generally, and will be found useful to executive officers whose duties comprise the supervision of our food supply. The author has dealt with the subject lucidly and is up to date with our present knowledge of the various diseases affecting fish, poultry, and also vegetables and fruit. The principles governing expert examination, and the methods of detection of unsoundness are enunciated. The reasons for the deterioration of food and the manner in which any departure from the normal may be expected to take place are also indicated. The book is capably illustrated from drawings made by the author himself, and concludes with a glossary of terms and a very clear index.

**The Sanitary Inspector's Handbook.** By HENRY H. CLAY. Pp. xx., 386. Illustrated. London: H. K. Lewis & Co. Ltd. 1933. Price 15s.—Practical handbooks by sanitary inspectors for sanitary inspectors are few, and when it was known that Mr. Clay had undertaken to replace Clarke's handbook with an up-to-date edition the publication was eagerly anticipated and the work eminently fulfils all expectation. The author was an expert sanitary inspector before he graduated to his present position on the staff of the London School of Hygiene and Tropical Medicine, and thus writes on a subject of which he has a masterly knowledge. The arrangement of the book is very good, commencing with an epitome of the evolution of the sanitary inspector's calling. The different phases of the inspector's duties are sub-divided, each chapter completing a particular subject. An important feature is that where the appropriate legal enactments have to be given they are interspersed with lucid explanatory notes, thus rendering the study of statutory powers not only easily readable but interesting. The illustrations, of which there are a large number, are clear and concise, showing the various methods of construction and the latest type of sanitary fittings. It has a very complete index. The work will rank as the standard text-book on the subject for the practising inspector as well as the student sanitary inspector.

**Stoke Park Monographs on Mental Deficiency.** No. 1. By RICHARD J. A. BERRY, M.D. Pp. xix., 249. Illustrated. London: Macmillan & Co. 1933. Price 10s. 6d.—The book is issued as a memorial to the late Rev. H. N. Burden, who founded and equipped at his own expense the National Institutions for Persons Requiring Care and Control, now housing nearly 2,000 patients. The full scope of the volume is not indicated by the title, as work is described carried out elsewhere than at Stoke Park. Some articles are for the student and others for the specialist. Of the articles published for the first time Berry and Norman describe the brain changes of three defectives. Norman contributes an interesting paper upon neurological findings. Cerebellar ataxia is described elaborately by Bates and Berry, but the excellent results of treatment are discussed too briefly. The observations recorded in the volume should prove of value to those with little knowledge of mental deficiency, but too much reliance must not yet be placed upon all the conclusions drawn from the observed facts. The volume is up to date and attractively produced, the illustrations are good, the references full and the index detailed.

**Psychology of Sex.** By HAVELOCK ELLIS. Pp. xii., 322. Illustrated. London: William Heinemann, Ltd. 1933. Price 12s. 6d.—The aim of this book is to present in concise form the essentials of a subject which enters into everyday practice and is receiving wider and wider attention. Routine medical teaching leaves the student almost entirely uninstructed in the bare elements of sex psychology. When he goes into practice he is at a loss when trying to deal with obvious problems in this field. The reason for this hiatus in medical education is clear, the subject-matter is unsuitable for ordinary bedside teaching. The book sets out in simple language the biological and social aspects of sex life from the psychological standpoint. The writer confines himself to facts disentangled from psychoanalytic theories. Particularly helpful is the chapter on marriage, which affords a basis for helpful advice and instruction to patients. Every practitioner will find in the book a great deal of information that he ought to know and a great deal that he can hand on to patients, when properly called upon to do so. Sympathy and common sense is wanted in a high degree in dealing with sex problems: there is little hope of achieving either in the absence of accurate knowledge of the subject. The book can be strongly recommended to both practitioner and student.

**Human Values in Psychological Medicine.** By C. P. Blacker, M.C., M.D., M.R.C.P. Pp. ix., 179. London: Oxford University Press. 1933. Price 8s. 6d.—Nowadays philosophy is concerning itself to an increasing extent with the subject of values. Heretofore medicine—even psychological medicine—has not concerned itself with this field of thought. A short review of the older philosophic conceptions of values is given, and Dr. Blacker then seeks to define his own conception of value. He thinks that it is to be derived from Macdougall's "essential and permanent nucleus of the instinct," and that the true basis of human valuation is to be found at the instinctive level and not in derived experience, though it may of course be modified by this. The significance of the individual is to be found in his idiosyncrasy, *i.e.* the particular "private mixture" of characteristics, especially mental, which differentiates him from his fellows. It must be remembered, however, that it is extremely difficult to describe such idiosyncrasies verbally, and the meanings used by psychologists are as a whole very difficult to convey in words at all.

In mankind values have been largely modified by socialization, but it might be expected that individuals will have individual "pivotal" values, *i.e.* that there will be something which is of major importance in their lives. The author has investigated this subject, and has found that his patients divide themselves into four groups: (1) Those who have no pivotal values, and are unconscious of ever having needed them; (2) those who have in fact pivotal values, but have never consciously needed them; (3) those who have needed such values, and have found them without much difficulty; (4) those who have consciously needed such values, and have had difficulty in finding them. The latter group tend to be neurotic. The author discusses various religious and philosophic valuations, and compares them to neurotic and psychotic reactions, and then goes on to discuss the difficult problem of reality at some length. This book undoubtedly contains much of interest, and the philosophical approach to psychiatric problems is unquestionably a valuable one. It is probable that many medical men, who are neither trained nor interested in philosophy, will be repelled by this very approach, but that is their misfortune and not the author's fault. None the less, there is a tendency to diffusion and following up of side-lines, no doubt interesting in themselves, but not strictly germane to the argument, which detracts from the value of the book as a serious contribution to psychopathological literature.

**Neurology—Psychiatry.** Practical Medicine Series. Edited by P. BASSOE, M.D., and F. G. EBAUGH, M.D. Pp. 471. Chicago: Year Book Publishers Inc. 1931.—This annual production is always to be welcomed. It consists of a series of articles from the medical literature of the world on Neurology and Psychiatry. Being an American publication, it is to be expected that American work looms most largely in the selection, but that is of advantage rather than of disadvantage to Neurologists and Psychiatrists working "on this side." The selection is comprehensive, but as inevitably happens, it is the frequent experience of the seeker for information that what he particularly wants is not there. However, if such subject-matter is not dealt with one year it almost certainly will appear another. The editorial comments introduced by Dr. Ebaugh in the psychiatric section are of definite interest and value. There can be no question that this and its companion volumes should be at the command of all serious students of the subject.

**Diseases of the Nervous System.** By W. RUSSELL BRAIN, D.M. Pp. xvi., 899. Illustrated. London: Oxford University Press. 1933. Price 27s. 6d.—The appearance of a new text-book of nervous diseases was needed at the present time. There have been a large number of important advances in neurology in

W

the last ten years which had yet to be incorporated in current text-books. The book is fully up to date, and the author seems to have left out nothing of importance. The classification of nervous disorders is always a matter of considerable difficulty. This is largely overcome by the preliminary survey of disorders of function in the light of anatomy and physiology, while in chapter vii there is an excellent review of a number of diseases from the ætiological standpoint. The reader will find that there is hardly any clinical problem which has escaped the attention of the author. Notably the book includes a useful chapter on diseases of the nervous system in relation to life insurance. An extension of this to a consideration of compensation cases would have been very helpful. The book can be strongly recommended for the use of the general practitioner and neurologist alike. The table of contents, index and bibliography are very complete and well arranged.

**A Synopsis of Surgery.** Tenth Edition. By ERNEST W. HEY GROVES, M.S., M.D., B.Sc. Pp. viii., 693. Illustrated. Bristol: John Wright & Sons, Ltd. 1933. Price 17s. 6d.—For twenty-five years this has been one of the outstanding successes amongst text-books for students, and it is not necessary to say much here to commend a book that has so thoroughly and deservedly established itself. The principal changes relate to the radium treatment of cancer, the Winnett Orr method of treating osteomyelitis, the surgery of the sympathetic nervous system, and amputations, all of which are excellent. Amid such a vast number of statements of fact and opinion there are, of course, some which would not command universal assent. A physician who chanced to read the section on Gastric Ulcer would be moved to indignation, and we must confess that we think the assertions made are too sweeping. The injection of A.B.A. might well be mentioned in the treatment of pruritus ani and fissure-in-ano, and radium for cancer of the thyroid. Is it not time that sarcoma disappeared from text-books amongst the alleged tumours of the breast? We are glad to see that Dr. Todd's lead selenide method of treating cancer is recognized.

**A Surgeon's Pocket Book.** By H. S. SOUTTAR, D.M., M.Ch. Pp. viii., 285. London: William Heinemann Ltd. 1933. Price 7s. 6d.—This work is actually a précis of Mr. Souttar's larger book on the subject of surgery, and a précis in its briefest possible form. It contains little more than headings and lists, brief notes, in fact, which the student should make for himself. While admitting the undoubted value of such a book, which is accurate to the last word, there must be the risk that it would, and could, replace that extraction and cutting down from a complete work on surgery and from lectures which is so valuable in impressing facts on the mind of the learner. The book can easily be carried in the pocket, and might well be of service during that last and terrible fortnight preceding an examination when we are in a perpetual state of suddenly remembering something we have forgotten to look up. If the book is to be used at all in the systematic study of surgery, it must be read in conjunction with a larger work on the subject and compared with the notes which the student makes for himself, for the purpose of correction and for the estimation of the comparative values of different methods of classification. Apart from its use for examination purposes, *A Surgeon's Pocket Book* might find favour in the eyes of the qualified man, whose memory is not all that he could wish.

**The Cure of Hæmorrhoids, Varicose Veins and Ulceration by Modern Methods of Injection and Bandaging.** By STUART McAUSLAND, M.D. Pp. vii., 63. Illustrated. London: John Bale, Sons and Danielsson Ltd. 1933. Price 3s. 6d.—This little volume comprises a description of all the modern treatments by sclerosing injections in a most lucid and succinct manner. The style makes easy reading, and yet there are many classifications which help the memory. In addition to discussing the pros and cons of the modern versus the old-established operative procedures, the author takes pains to define the nature of the minor maladies which the methods are intended to alleviate. In this way he protects both the doctor and patient from the pitfall of treating for piles when this condition is perhaps only a secondary subsidiary manifestation of cancerous or other serious trouble in the recto-colon. It is pre-eminently a practical guide. The procedure and diagnosis of each step in the treatment is described in every detail, so that the amateur may quickly attain the skill of the expert. The illustrations are numerous and clear.

**Practical Points in Eye Surgery and Dressing.** By HUGH E. JONES, M.R.C.S. Pp. 27. London: John Bale, Sons & Danielsson Ltd. 1933. Price 2s. 6d. This small volume is addressed to nurses, house surgeons and general practitioners, the author's main objects being to assist them in the management of eye cases until expert advice can be obtained, and in the preparation, after-treatment, etc., of patients requiring operation. He provides a great deal of sound practical information in a handy form for ready reference. Three tables, contained in an envelope in the cover, are especially useful. They deal with the differential diagnosis and immediate treatment of urgent non-traumatic cases and of injuries, instruments used and drops required before and after operation, patients' diet and duration in bed, the admission of visitors, etc. The instructions are concise and necessarily rather dogmatic, but realizing that other surgeons may favour variations in some of the details, the author has provided space for additional notes. To those without special experience in the subject the book can be recommended as a most useful guide when ophthalmic problems have to be solved.

**Natural Childbirth.** By G. D. READ, M.D. Pp. ix., 127. Illustrated. London: William Heinemann Ltd. 1933. Price 7s. 6d.—This interesting little book discusses the problems of childbirth from a novel angle. The psychological and emotional aspects of labour are presented in a most helpful manner. The author has performed a most useful service in stressing the importance of fear as a very real factor in the causation of difficulty in childbirth. All who have to do with obstetric practice will profit by reading this useful and stimulating work.

**The Intervertebral Discs.** By ORMOND A. BEADLE. A special report issued by the Medical Research Council. Pp. 79, Figs. 47. London: H.M. Stationery Office. Price 2s.—For many years past Professor Schmorl in Dresden has made an intensive study of the anatomy and pathology of the spine, with especial regard to static deformities. Over 7,000 spines have been removed post-mortem and sectioned, and over 600 of these put up as permanent museum specimens. As a result of all this patient investigation there has come to our knowledge the facts relating to the variations to which the intervertebral discs are subject, both in health and disease. The most remarkable of these abnormalities is the enlargement of the central part of the pulpy disc and its fungation, as it were, into the cancellous tissue of the adjacent vertebræ. The author of the present monograph has done a great service to English readers by putting together in a convenient form these researches of Schmorl. Of very great value is the series of forty-seven plates, which give a beautiful representation of the naked-eye and microscopic anatomy of the spine and its discs.

**An Outline of Medicine for Nurses.** By JAMES FARMING, M.D., D.P.H. Pp. 136. Bristol: John Wright & Sons, Ltd. 1933. Price 2s. 6d. — This small book is essentially a condensation product. It contains a large amount of useful information well presented ; but necessarily suffers from some of the defects associated with an attempt to include too much in a small space. The chapter on heart disease in particular appears inadequate and too brief to be useful. A similar criticism applies to the section on anæmia, particularly pernicious anæmia, which contains no mention of subacute combined degeneration or achlorhydria. Respiratory diseases are much better done, though a notable omission is found in the absence of any reference to the treatment of hæmoptysis. Alimentary disorders are well done. There is a useful chapter on infant feeding and on the dietary of children, though four-hourly breast feeding is not mentioned. Renal and nervous diseases are adequately treated in the small space available. The book is useful as a guide to anyone who is called upon to lecture to nurses and who has to pay attention to the standard syllabus.

**Neurological Effects of Syphilis.** By B. B. SHARP, M.D. Pp. viii., 92. Illustrated. London : Oxford University Press. 1933. Price 7s. 6d.—This little volume contains an interesting and readable account of neurosyphilis, in its pathological and therapeutic aspects. Most of the modern work on the morbid anatomy has been incorporated. A detailed description is given of the various lines of treatment, but enthusiasm for new methods seems to have squeezed out any recognition of mercury as a therapeutic agent. The book is entirely a compilation and bears no trace of original observation or research work ; there is, moreover, a paucity of critical comment. Its object and scope are not easy to determine.

